# ﻿*Kelawakaju* gen. nov., a new Asian lineage of marpissine jumping spiders (Araneae, Salticidae, Marpissina)

**DOI:** 10.3897/zookeys.1130.87730

**Published:** 2022-11-17

**Authors:** Wayne P. Maddison, Gustavo R. S. Ruiz, Paul Y. C. Ng, Ettukandathil Haridas Vishnudas, Ambalaparambil V. Sudhikumar

**Affiliations:** 1 Departments of Zoology and Botany and Beaty Biodiversity Museum, University of British Columbia, 6270 University Boulevard, Vancouver, British Columbia, V6T 1Z4, Canada University of British Columbia Vancouver Canada; 2 Instituto de Ciências Biológicas, Universidade Federal do Pará, Rua Augusto Corrêa, 01, CEP 66075-110, Belém, PA, Brazil Universidade Federal do Para Belém Brazil; 3 205 River Valley Road, #16-53, Singapore 238274, Singapore Unaffiliated Singapore Singapore; 4 Centre for Animal Taxonomy and Ecology, Department of Zoology, Christ College, Irinjalakuda, Kerala, 680 125, India Christ College Irinjalakuda India

**Keywords:** Classification, Dendryphantini, molecular phylogeny, new genus, new species, Salticinae, Salticoida, taxonomy

## Abstract

The genus *Kelawakaju* Maddison & Ruiz, **gen. nov.**, is described for a lineage of bark-dwelling Asian marpissine jumping spiders that represent a dispersal to Eurasia separate from that of the *Marpissa*-*Mendoza* lineage, according to the phylogeny recovered from analysis of four gene regions. All species of *Kelawakaju* are new to science except *Kelawakajufrenata* (Simon, 1901), **comb. nov.**, which is transferred from *Ocrisiona* Simon, 1901. *Kelawakajufrenata* is known from Hong Kong, Guangdong, Guangxi, and likely Taiwan. The five new species are *Kelawakajumulu* Maddison & Ruiz, **sp. nov.** (type species of *Kelawakaju*, from Sarawak, Malaysia, ♂♀), *K.intexta* Maddison & Ruiz, **sp. nov.** (from Sarawak, ♂), *K.leucomelas* Maddison & Ng, **sp. nov.** (Singapore and Johor Bahru, ♂♀), *K.sahyadri* Vishnudas, Maddison, & Sudhikumar, **sp. nov.** (India, ♂♀), and *K.singapura* Maddison & Ng, **sp. nov.** (Singapore, ♂♀).

## ﻿Introduction

Jumping spiders of the tribe Dendryphantini diversified into more than 700 known species largely in the Americas (Maddison 2015), but a few lineages reached the Old World: a few genera in the Dendryphantina, one genus in the Synagelina, and two genera in the Marpissina. The two marpissine genera, *Marpissa* C. L. Koch, 1846 and *Mendoza* Peckham & Peckham, 1894, are similar and likely closely related (Logunov 1999), possibly representing a single dispersal into the Palearctic. There is, however, another distinct lineage of the Marpissina in Asia, hidden taxonomically because its one described species has been misplaced to genus and tribe. Simon (1901b) chose the astioid genus *Ocrisiona* Simon, 1901 as the home for his species *O.frenata* Simon, 1901, described from Hong Kong. The type species and others of the primarily Australasian *Ocrisiona* (Astioida: Viciriini) are elongate and flat-bodied, as is *O.frenata*, but the latter species is a marpissine rather than an astioid, as we show here. Field work has revealed that *O.frenata* is not alone but is part of a small radiation of tree trunk dwelling marpissines in tropical Asia. We here describe the new genus *Kelawakaju*, gen. nov., to contain *K.frenata*, comb. nov., and five new species.

## ﻿Materials and methods

Spider specimens examined for this study are stored in the University of British Columbia Spencer Entomological Collection, Canada (**UBCZ**), the Lee Kong Chian Natural History Museum, Singapore (**LKCNHM**, https://lkcnhm.nus.edu.sg), the Research Collections at National Centre for Biological Sciences, Bengaluru, Karnataka, India (**NCBS**, http://biodiversitycollections.in), and the Centre for Animal Taxonomy and Ecology, Christ College, Thrissur, Kerala, India (CATE).

Preserved specimens were examined under both dissecting microscopes and a compound microscope with reflected light. Drawings were made with a drawing tube on a Nikon ME600L compound microscope. Most photographs of living specimens were made with either a Pentax Optio 33WR digital camera with a small lens glued to it for macro capability or an Olympus OM-D E-M10 II camera with 60 mm macro lens.

All measurements are given in millimeters. Descriptions of color pattern are based on the alcohol-preserved specimen. Carapace length was measured from the base of the anterior median eyes not including the lenses to the rear margin of the carapace medially; abdomen length to the end of the anal tubercle. The following abbreviations are used: **PLE**, posterior lateral eyes; **RTA**, retrolateral tibial apophysis.

Molecular data was gathered for four gene regions by traditional Sanger PCR methods and combined with previously published data to compose a dataset of 36 taxa (Table 1) including 32 species of marpissoids (14 Marpissina, 3 Itatina, 8 Dendryphantina, 4 Synagelina, 3 Ballini) and 4 outgroups (1 Plexippini, 1 Baviini, 2 Astioida). Preservation, DNA extraction, PCR, and sequencing of nuclear 28S and Actin 5C and mitrochondrial 16SND1 and COI followed the protocols of Zhang and Maddison (2013) and Maddison et al. (2014). Alignments were done by MAFFT with the L-INS-i option (Katoh and Standley 2013), with edges of coding regions of Actin and ND1 refined by hand using amino acid translation in Mesquite 3.61 (Maddison and Maddison 2021) and comparison to sequences with known boundaries. The Actin intron aligned so poorly that it was excluded entirely from phylogenetic analyses (Maddison et al. 2014).

Maximum-likelihood phylogenetic analyses were performed with IQ-TREE version 1.6.7.1 (Nguyen et al. 2015) using the Zephyr 3.1 package (Maddison and Maddison 2020) in Mesquite 3.7 (Maddison and Maddison 2021). The four genes were concatenated and set into seven partitions expected to have potentially different models of evolution: 28S, 16S (including other non-coding parts of 16SND1), mitochondrial codon positions 1 and 2, mitochondrial codon position 3, Actin codon position 1, Actin 2, and Actin 3. IQ-TREE was run with the options -m TESTMERGE -spp to allow the partitions to be merged and their models chosen according to the Bayesian information criteria. (The best partition scheme united Actin 1 and 2 to yield six partitions, with models 28S: TIM3+F+I+G4, 16S: GTR+F+I+G4, mitochondrial 1, 2: TIM2+F+I+G4, mitochondrial 2: HKY+F+I+G4: Actin 1 + 2: SYM+I, Actin 2: JC, Actin 3: K3Pu+F+G4.) The maximum likelihood tree was sought with 50 search replicates, and repeatability assessed with 1000 standard bootstrap replicates.

Alignments and trees are deposited in the Dryad data repository (http://dx.doi.org/10.5061/dryad.mw6m9060r).

## ﻿Results

### ﻿Molecular phylogenety

**Figure 1.**
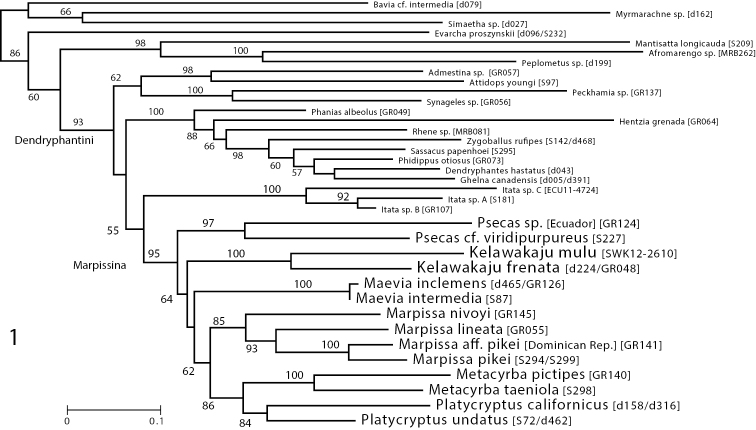
Maximum likelihood phylogeny of Dendryphantini showing placement of *Kelawakaju* species as a distinct lineage in the Marpissina. Based on 28S, Actin 5C, 16SND1, COI gene regions; numbers indicate percentage of 1000 bootstrap replicates showing clade.

The reconstructed phylogeny (Fig. 1) gives support for *Kelawakaju* being a monophyletic group within the Marpissina, and distinct from other genera, including the *Marpissa*, another known Eurasian marpissine. As expected, the Dendryphantini and each of its subtribes are monophyletic. The two species of *Kelawakaju* are monophyletic together, distinct from described marpissine genera, and placed as a relatively deep branching lineage in the Marpissina, although the bootstrap support is not high. These results suggest that marpissines dispersed from the Americas (where most marpissoid diversity lies; Maddison 2015) into the Old World at least twice, once for *Marpissa-Mendoza*, and once for *Kelawakaju*.

The phylogenetic results emphasize the difficulties faced in recognizing salticid relationships from general appearances. When one author (WPM) first collected members of the *K.mulu* group, he recorded them as baviines, and assumed that their resemblance to the marpissine *Balmaceda* Peckham & Peckham, 1894 was convergence for trunk-dwelling. It was only with the molecular data that their identity as marpissines became clear. When other authors (EHV, AVS) first collected *K.sahyadri*, they also thought it likely to be a baviine. Simon (1901a, b) considered *K.frenata* congeneric with the viciriine *Ocrisiona*. It is indeed easy to confuse various marpissines, baviines, viciriines, and bredines, for convergence has given them similar body forms.

**Table 1.** Specimens and GenBank accession numbers of four gene regions analyzed. Accession numbers with * indicate already published (Hedin and Maddison 2001; Maddison and Hedin 2003; Maddison and Needham 2006; Maddison et al. 2007, 2008, 2014; Vink et al. 2008; Bodner and Maddison 2012; Ruiz and Maddison 2015; Maddison 2016; Maddison and Szűts 2019).

Species	Specimen ID	Locality	28S	Actin	16SND1	CO1
**Non-marpissoid outgroups**						
*Evarchaproszynskii* Marusik & Logunov, 1998	d096/S232	Canada: British Columbia	DQ665765*	EU522704*	DQ665723*	AY297379*
Baviacf.intermedia (Karsch, 1880)	d079	Malaysia: Sabah	EU815490*	KM032958*	KM032925*	EU815603*
*Myrmarachne* sp.	d162	Malaysia: Pahang	EU815507*	JX145837*	EU815565*	EU815616*
*Simaetha* sp.	d027	Australia: Queensland	EU815477*	JX145839*	EU815546*	EU815592*
**Ballini**						
*Afromarengo* sp.	MRB262	Gabon: Ngounié: Waka National Park	JX145758*	JX145842*	JX145905*	JX145682*
*Mantisattalongicauda* Cutler & Wanless, 1973	S209	Philippines: Luzon	AY297270*		AY296689*/AY297333*	AY297399*
*Peplometus* sp.	d199	Ghana: N. of Cape Coast, Kakum Forest	EU815515*	JX145843*	EU815572*	EU815621*
**Dendryphantini: Synagelina**						
*Admestina* sp.	GR057	U.S.A.: Mississippi	OP605970	OP700690	OP700674	
*Attidopsyoungi* (Peckham & Peckham, 1888)	S97	U.S.A.: Missouri	AF327933*		AF327961*/AF328020*	AF327990*
*Peckhamia* sp.	GR137	Dominican Republic: Barahona	OP605980	OP700699	OP700683	
*Synageles* sp.	GR056	U.S.A.: Mississippi	OP605985	OP700705	OP700689	
**Dendryphantini: Dendryphantina**						
*Dendryphanteshastatus* (Clerck, 1757)	d043	Poland: Siedlce	EF201646*	KY200848*	KM032927*	KM033228*
*Ghelnacanadensis* (Banks, 1897)	d005	U.S.A.: North Carolina	EF201651*	EU522708*	OP700675	
	d391	U.S.A.: North Carolina				KT462689
*Hentziagrenada* (Peckham & Peckham, 1894)	GR064	USA: Florida	OP605971	OP700691	OP700676	
*Phaniasalbeolus* (Chamberlin & Ivie, 1941)	GR049	Canada: British Columbia	OP605981	OP700700	OP700684	
*Phidippusotiosus* (Hentz, 1846)	GR073	USA: Florida	OP605982	OP700701	OP700685	
*Rhene* sp.	MRB081	China: Guangxi	OP605984	OP700704	OP700688	
*Sassacuspapenhoei* Peckham & Peckham, 1895	S295	U.S.A.: Arizona	AF327953*		AF327982/AF328041*	AF328012*
*Zygoballusrufipes* Peckham & Peckham, 1885	S142	U.S.A. and Panama	AF327944*		AF327972*/AF328031*	AF328002*
**Dendryphantini: Itatina**						
*Itata* sp. A	S181	Ecuador: Manabi	AF327932*		AF327960*/AF328019*	AF327989*
*Itata* sp. B	GR107	Ecuador: Napo	OP605972	OP700692		
*Itata* sp. C	ECU11-4724	Ecuador: Orellana:Yasuní	OP605973		OP700677	
**Dendryphantini: Marpissina**						
*Kelawakajumulu* sp. nov.	SWK12-2610	Malaysia: Sarawak: Mulu	OP605974		OP700678	OP606004
*Kelawakajufrenata* (Simon, 1901)	d224	China: Guangxi	JX145769*		JX145911*	JX145688*
	GR048	China: Guangxi		OP700693		
*Maeviainclemens* (Walckenaer, 1837)	d465	USA: Tennessee		OP700694		
	GR126	USA: North Carolina	OP605975			
*Maeviaintermedia* Barnes, 1955	S87	USA: Alabama	AY297269*		AY296688*/AY297332*	AY297398*
*Marpissalineata* (C. L. Koch, 1846)	GR055	USA: Mississippi	OP605977	OP700696	OP700680	
*Marpissanivoyi* (Lucas, 1846)	GR145	Spain: Sitges	OP605978	OP700697	OP700681	
*Marpissapikei* (Peckham & Peckham, 1888)	S294/S299	USA: Arizona	AF327936*		AF327964*/AF328032*	AF327993*
Marpissaaff.pikei (Peckham & Peckham, 1888)	GR141	Dominican Republic: Pedernales	OP605976	OP700695	OP700679	
*Metacyrbapictipes* Banks, 1903	GR140	Dominican Republic: Pedernales	OP605979	OP700698	OP700682	
*Metacyrbataeniola* (Hentz, 1846)	S298	USA: Arizona	AY297271*		AY296690*/AY297334*	
*Platycryptuscalifornicus* (Pkm & Pkm, 1888)	d316	Canada: British Columbia	KM033194*	KM032960*		KM033229*
	d158	Canada: British Columbia			OP700686	
*Platycryptusundatus* (De Geer, 1778)	S72	U.S.A.: Florida	AF327935*		AF327963*/AF328022*	AF327992*
	d462	Canada: Ontario: St. Williams		OP700702		
Psecascf.viridipurpureus (Simon, 1901)	S227	Ecuador: Sucumbios	AY297273*		AY297336*	AY297400*
*Psecas* sp.	GR124	Ecuador: Napo	OP605983	OP700703	OP700687	

### ﻿Taxonomy

#### ﻿ Kelawakaju

Taxon classificationAnimaliaAraneaeSalticidae

Maddison & Ruiz gen. nov.

10D59B32-B7EC-564F-AEB3-67B515C5B0D0

https://zoobank.org/1A91FAF6-5C6F-4AAB-A770-2395DE6CCAF3

##### Type species.

*K.mulu* Maddison & Ruiz, sp. nov.

##### Species included.

*K.mulu* species group:

*Kelawakajumulu* Maddison & Ruiz, sp. nov.

*Kelawakajuintexta* Maddison & Ruiz, sp. nov.

*K.singapura* species group:

*Kelawakajusingapura* Maddison & Ng, sp. nov.

*K.frenata* species group:

*Kelawakajufrenata* (Simon, 1901)

*Kelawakajuleucomelas* Maddison & Ng, sp. nov.

*Kelawakajusahyadri* Vishnudas, Maddison, & Sudhikumar, sp. nov.

##### Etymology.

The name means tree spider in the Berawan language from the area of Long Terawan, Sarawak (*kelawak* = spider; *kaju* or *kajuh* = tree; Syria Lejau Malang, pers. comm.), where the first specimens of *K.mulu* were found. To be treated grammatically as feminine.

##### Diagnosis.

Elongate and flat-bodied salticids, unusual among marpissines for the elongated or enlarged male chelicerae. Retrolateral tibial apophysis of palp long, blade-like, more or less straight and parallel to axis of palp. Embolus relatively short among marpissines, arising more or less terminally on the bulb (9–12 o’clock in ventral view of left palp). Markings cryptic on tree trunks, either mottled or with low-contrast longitudinal bands.

##### Description.

**Figures 2–9.**
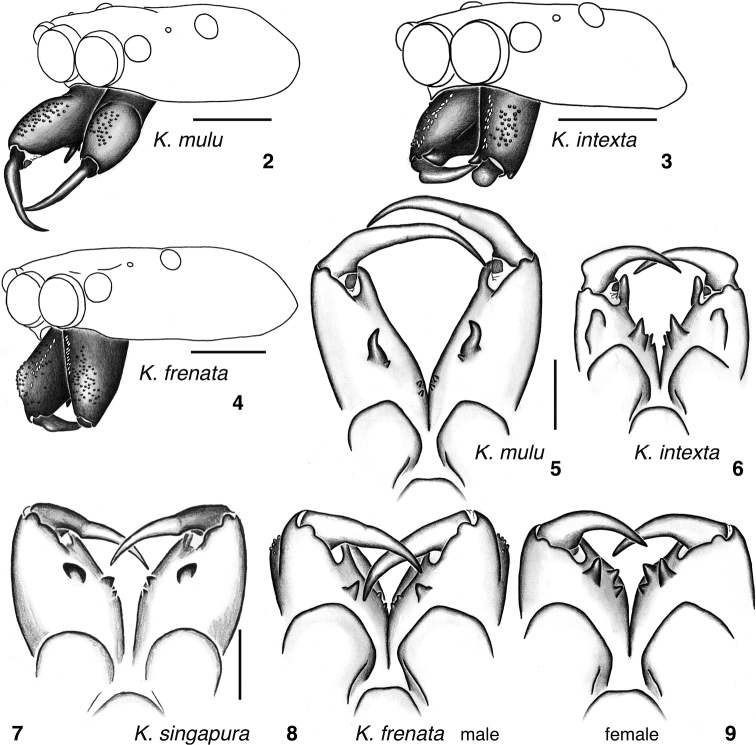
Chelicerae of *Kelawakaju* species **2–4** oblique view with carapace **5–9** ventral view **2***K.mulu* male holotype **3***K.intexta* male holotype **4***K.frenata* male from Guangxi **5***K.mulu* male holotype **6***K.intexta* male holotype **7***K.singapura* male holotype **8***K.frenata* male from Guangxi **9***K.frenata* female from Guangxi. Scale bars: 1.0 mm.

**Figures 10–15.**
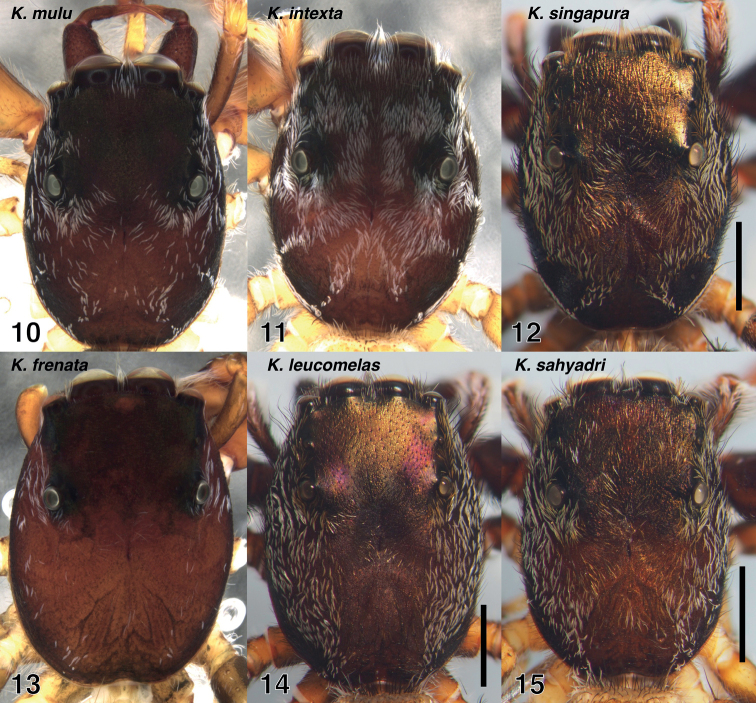
Carapaces of *Kelawakaju* males **10***K.mulu* holotype **11***K.intexta* holotype **12***K.singapura* holotype **13***K.frenata* from Guangxi, Dongxing **14***K.leucomelas* holotype **15***K.sahyadri* holotype. Scale bars: 1.0 mm.

***Carapace*** flat, narrower (*K.mulu* group, Figs 10, 11) to broader (*K.leucomelas*, Fig. 14). Lower part of the thorax in some species with 1–3 distinct narrow vertical lines of pale scales (*K.mulu*: Fig. 27; *K.intexta*: Fig. 32; *K.singapura*: Figs 43, 45), resembling similar stripes in the baviine *Piranthus* Thorell, 1895 (Maddison et al. 2020: fig. 263) and the gophoine *Cotinusa* Simon, 1900 (Rubio and Baigorria 2016). ***Chelicerae*** with seta-bearing tubercles on paturon of males and some females (Figs 2–4). Males of all but two species have narrow stripes of white scales on the front face of the chelicerae, forming an inverted V (Figs 3, 4, 74, 79, 83). Two promarginal teeth and one retromarginal tooth (sometimes with a second cusp, Fig. 6). ***Palp’s***RTA a long blade. Embolus appears freely movable, separate from functional tegulum. Cymbium modified at ventral-retrolateral-proximal corner (e.g., Figs 17, 21, 47). ***Abdomen*** long and narrow.

We recognize three species groups in the genus.

###### *Kelawakajumulu* species group

The *mulu* species group includes *K.mulu*, *K.intexta*, and a third as-yet-undescribed species from Singapore. They are smaller-bodied than other *Kelawakaju*, with mottled markings, and narrow chelicerae that project forward in the male. The embolus is narrow and forms a smooth curve bending toward the retrolateral. The lower part of the thorax has three vertical stripes of pale scales on each side. Epigynal openings are delicate and the edges difficult to discern (Fig. 18). Retromarginal tooth of chelicera with small second cusp basally (Figs 5, 6). Members of this group may prefer more shaded habitats than those of the *frenata* group, having been found only inside forests.

#### ﻿ Kelawakaju mulu

Taxon classificationAnimaliaAraneaeSalticidae

Maddison & Ruiz sp. nov.

550365B6-A729-5A31-8C2A-2698351441AD

https://zoobank.org/C1730DAC-227A-4384-B25D-CBDD46B37E76

Figs 2 , 5 , 10 , 16–20 , 23–28

##### Type material.

***Holotype***: male (SWK12-2610) in UBCZ from Malaysia: Sarawak: Mulu Nat. Pk., Summit Trail near Camp 1, 4.0486°N, 114.8610°E to 4.0483°N, 114.8614°E, 270 m elev., 21 March 2012, Maddison/Piascik/Ang WPM#12-072. ***Paratype***: female (SWK12-2639) in UBCZ from Malaysia: Sarawak: Mulu Nat. Pk., Summit Trail near Camp 1, 4.0480°N, 114.8626°E to 4.0478°N, 114.8630°E, 290–320 m elev., 22 March 2012, Piascik/Ang/Andyson WPM#12-077.

##### Etymology.

From the name of the type locality (a noun in apposition).

##### Diagnosis.

**Figures 16–22.**
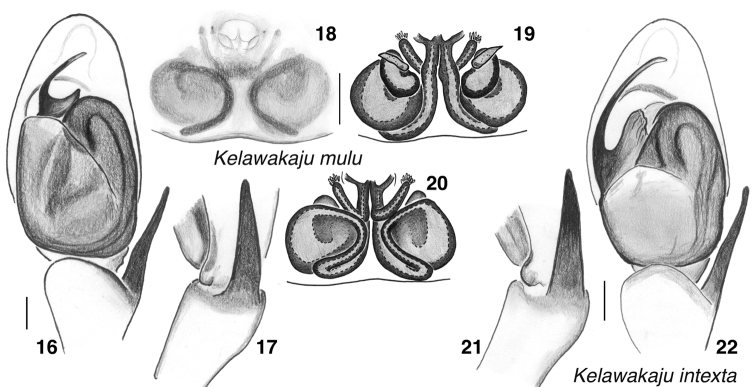
*Kelawakajumulu* species group, genitalia **16–20***K.mulu***16** holotype male palp, ventral **17** same, retrolateral **18** paratype female SWK12-2639 epigyne, ventral **19** same, vulva, dorsal **20** same, ventral **21, 22***K.intexta* holotype male palp **21** retrolateral **22** ventral. Scale bars: 0.1 mm.

**Figures 23–28.**
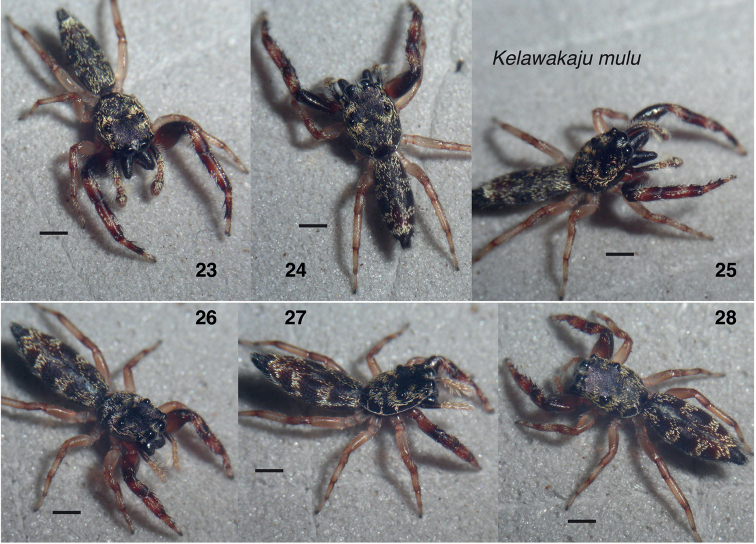
*Kelawakajumulu***23–25** holotype male SWK12-2610 **26–28** paratype female SWK12-2639. Scale bars: 1.0 mm.

Dark with only a dusting of golden scales, unlike the similar but more thoroughly scale-covered *K.intexta* (Figs 23–28 vs. 29–34). Embolus shorter than that of *K.intexta*, arising at 11 o’clock (Figs 16 vs. 22).

##### Description.

**Male** (based on holotype). Carapace length 2.85; abdomen length 3.05. ***Carapace*** dark brown, with white scales around cephalic region, between AME and sparse on thoracic region. ***Clypeus*** very narrow. ***Chelicera*** dark brown, elongate and projected, with a line of white scales on the prolateral face. Retromarginal tooth with two cusps, the more lateral long and curved (Fig. 5). ***Palp*** with elongate RTA. Embolus narrow and curved, but short, arising distally on the tegulum. Endite subrectangular, with no projection, dark brown. Labium dark brown and sternum light brown, with depressions along coxae I. ***Leg*** I light brown, with mid patella, mid tibia, proximal area of metatarsus and entire tarsus yellow. Legs II–IV yellow. Length of femur I 2.10, II 1.70, III 1.40, IV 1.65; patella + tibia I 3.10, II 2.40, III 1.65, IV 2.35; metatarsus + tarsus I 1.85, II 1.75, III 1.50, IV 1.70. Leg spination reduced: femur I d0, p0-0-1-0 (or p0-0-2-0), II d1-1-0, p0-0-1-0, III 0, IV d1-1-0, r0-0-1-0; patella I–IV 0; tibia I v2-2-2 (asymmetrical), II v1r-1r-1p, III–IV 0; metatarsus I–II v2-2, III 0, IV v0-0-1p. ***Abdomen*** dorsally dark brown, with two transverse wide light stripes, and a third over anal tubercle; ventrally gray.

**Female** (based on paratype SWK 12-2639). Carapace length 2.7; abdomen length 3.55. Color as in male, except when mentioned. ***Chelicera*** light brown. Retromarginal tooth with two cusps, the distal one almost twice the size of the other, both acute. ***Legs*** II–IV with narrow stripes of white scales. Length of femur I 1.90, II 1.50, III 1.45, IV 1.85; patella + tibia I 2.60, II 1.90, III 1.75, IV 2.60; metatarsus + tarsus I 1.40, II 1.30, III 1.60, IV 1.90. Leg spines as in male, except for femur III, as in II. ***Abdomen*** as in male, except for stripes, medially interrupted; ventrally white, with two longitudinal dark brown stripes extending from booklungs to spinnerets. ***Epigyne*** with a pair of small copulatory openings distant from the posterior border, which has a medial excavation; internally, copulatory ducts fuse with glandular portions, spiral backwards and enter the large spermathecae, from which fertilization ducts emerge.

##### Natural history.

Both specimens were collected on tree trunks on a forested slope.

#### ﻿ Kelawakaju intexta

Taxon classificationAnimaliaAraneaeSalticidae

Maddison & Ruiz sp. nov.

B65D2876-F70E-579E-8292-9BA84F1D8537

https://zoobank.org/58AE0276-1E71-45DA-AAFB-EAD822AE60F3

Figs 3 , 6 , 11 , 21 , 22 , 29–34

##### Type material.

***Holotype***: male (SWK12-3752) in UBCZ from MALAYSIA: Sarawak: Lambir Hills Nat. Pk., headquarters area, 4.197 to 4.198°N 114.0400°E to 114.0402°E, 50 m elev., 30 March to 6 April 2012 Maddison/Piascik/Ang WPM#12-104. ***Paratype***: male (SWK12-0523) in UBCZ from MALAYSIA: Sarawak: Bako Nat. Pk. Ulu Assam Trail, 1.712°N, 110.445°E to 1.713°N, 110.448°E, 30–80, m elev., 8 March 2012, Maddison/Piascik/Ang/Lee WPM#12-005.

##### Etymology.

Latin, interwoven, referring to the textile-like pattern of coloured scales on the body.

##### Diagnosis.

**Figures 29–34.**
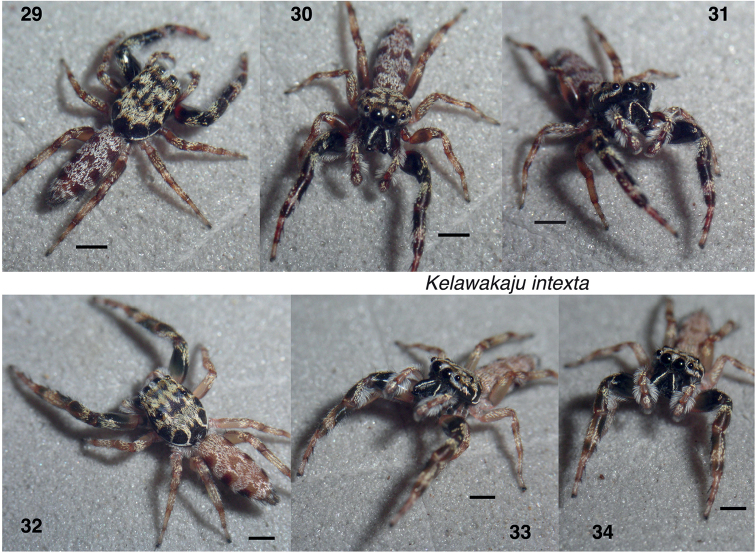
*Kelawakajuintexta***29–31** holotype male SWK12-3752 **32–34** paratype male SWK12-0523. Scale bars: 1.0 mm.

Body covered with a dense and intricate pattern of pale scales, white on the abdomen and slightly golden on the carapace (Figs 29, 32), and thus paler in appearance than *K.mulu*. Embolus arising at 9 to 10 o’clock, longer than in any other *Kelawakaju* (Fig. 22).

##### Description.

**Male** (based on holotype). Carapace length 2.45; abdomen length 3.45. ***Carapace*** dark brown, with white scales on cephalic region, sparse on thoracic region and with line of white scales along borders of carapace. ***Clypeus*** very narrow. ***Chelicera*** dark brown, slightly projected, with mastidion. Retromarginal tooth with two cusps, the more lateral larger (Fig. 6). ***Palp*** light brown. RTA elongate. Embolus narrow, gently curving from its base, longer than half the length of the tegulum, arising prolaterally from the tegulum. Endite dark brown. Labium dark brown and sternum light brown. ***Leg*** I dark brown, with proximal portion of femur, mid tibia and metatarsus light brown, and tarsus yellow; II–IV light brown. Length of femur I 2.10, II 1.70, III 1.40, IV 1.65; patella + tibia I 3.10, II 2.40, III 1.65, IV 2.35; metatarsus + tarsus I 1.85, II 1.75, III 1.50, IV 1.70. Leg spination reduced: Femur I–II d1-1-0, p0-0-1, III d1-1-1, p0-0-1, IV d1-1-1, r0-0-1, patella I–IV 0, tibia I v2-2-2, II v1r-1r-1p, III–IV 0, metatarsus I–II v2-2, III 0, IV v0-0-1p. ***Abdomen*** dorsally with three pairs of dark marks with dark scales, among light areas with white scales; entirely covered by scutum; ventrally gray, with dark brown ring around spinnerets. Spinnerets yellow.

**Female** unknown.

##### Natural history.

The paratype from Bako was found along a trail in a forest.

###### *Kelawakajusingapura* species group

The *singapura* species group includes only *K.singapura*, distinctive for the robust male chelicerae, short and stout embolus, and the long palp tibia (longer than the tibial apophysis). It is larger-bodied, like the *frenata* group, but has a longer ocular quadrangle, and the abdominal markings are inverted compared to the *frenata* group: dark laterally, paler medially, similar to *K.intexta* of the *mulu* group. There is no clear indication to which of the other two groups *K.singapura* is more closely related, and hence we keep it separate.

#### ﻿ Kelawakaju singapura

Taxon classificationAnimaliaAraneaeSalticidae

Maddison & Ng sp. nov.

1DA11772-C36D-5638-8466-03F7D8B6F02B

https://zoobank.org/418101EA-5EED-4C48-87C7-5411F4FDF216

Figs 7 , 12 , 35–45

##### Type material.

***Holotype***: male (JK.21.08.02.0001) in LKCNHM from Singapore: Labrador Nature Reserve, 1.2653°N, 103.8019°E, J.K.H. Koh & P.Y.C. Ng, 2 August 2021. ***Paratypes***: One female (JK.21.05.14.0001) in LKCNHM from Singapore: Labrador Nature Reserve, 1.2664°N, 103.8014°E, J.K.H. Koh & P.Y.C. Ng, 14 May 2021. One male (90.10.21.0002) in LKCNHM from Singapore: Simpang, 1.44°N, 103.85°E, J.K.H. Koh, 21 October 1990. One female (AS19.0023) in UBCZ from Singapore: Adam Road, 1.336°N, 103.816°E, 10 m elev., 1–2 June 2019, W. Maddison & P.Y.C. Ng WPM#19-030.

##### Etymology.

From name of the type locality, Singapura in the Malay language, a noun in apposition.

##### Diagnosis.

**Figures 35–45.**
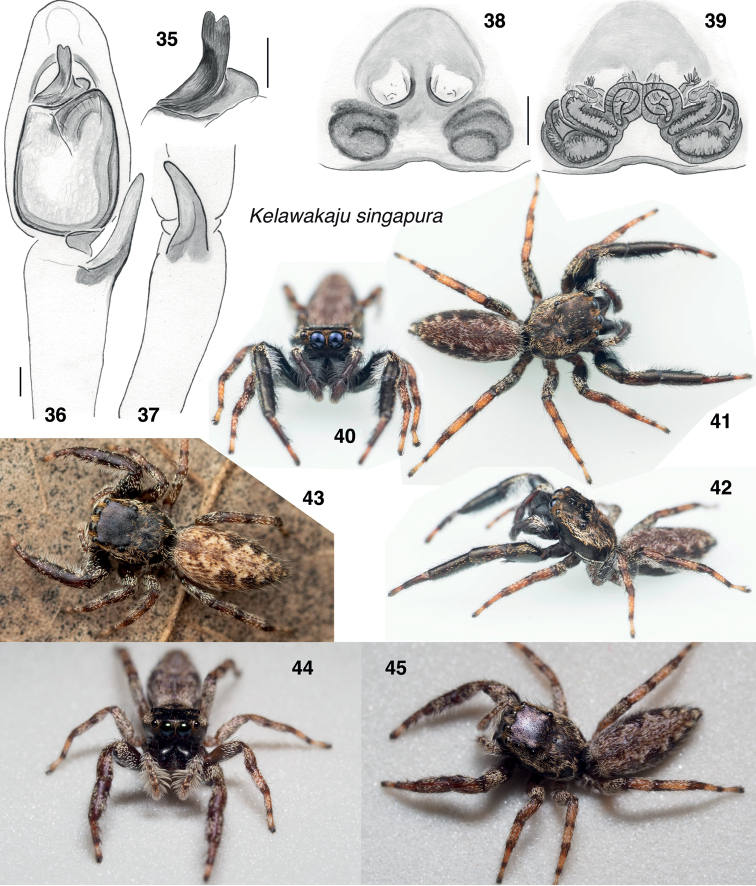
*Kelawakajusingapura***35** holotype male JK.21.08.02.001 embolus, ventral **36** paratype male JK.90.10.21.0002 palp, ventral **37** same, retrolateral **38** paratype female AS19.0023 epigyne, ventral **39** same, vulva, dorsal **40–42** holotype male **43** paratype female JK.21.05.14.0001 **44, 45** paratype female AS19.0023. Scale bars: 0.1 mm.

Distinctive for the abdomen’s central pale longitudinal band with wavy edge (Figs 41, 43, 45), short and stout embolus (Figs 35, 36), long tibia on the male palp (Figs 36, 40), and broad rounded retromarginal tooth on the male chelicera (Fig. 7). The male’s chelicerae are relatively more robust than in other species, which in contrast have narrower and more projecting or diverging chelicerae.

##### Description.

**Male** (based on holotype). Carapace length 3.1, width 2.3; abdomen length 3.9. ***Carapace*** (Figs 12, 41, 42): Distinctly wider just behind PLE. Depressed around fovea. Integument black to very dark brown. Thorax with dark setae near lower margin, interrupted by a fine vertical line of pale scales on each side at posterior corner; upper thorax clothed with pale scales; a few scales in ocular quadrangle. Narrow band of white scales along margin posterior to PLE. ***Clypeus*** narrow, dark, with black hairs. ***Chelicerae*** projecting only slightly, robust. Dark brown, with black hairs, many of which arise from tubercles. Retromarginal tooth a broad rounded flange, broadening from base. ***Palp*** tibia long. Embolus erect but short, broad, bifid at tip (Fig. 35). Integument black to brown, with black setae except white setae on last third of femur. Long black setae project laterally along length of tibia (not as a narrow brush). Endite subrectangular, with no projection, dark brown. ***Legs*** medium to dark brown. First leg dark brown except slightly paler at base of patella, which has white setae, and the honey-coloured tarsus. Patella with some white scales above and fringe of white hairs below, which continues onto the distal portion of the femur. Posterior legs with somewhat annulate markings. Length of femur I 2.0, II 1.5, III 1.5, IV 1.6; patella + tibia I 3.0, II 2.1, III 1.7, IV 2.5; metatarsus + tarsus I 1.8, II 1.4, III 1.6, IV 1.8. Leg spination reduced: femur I d0-1-0 (or 0-1-1), p0-2-0, II d1-1-1, p0-0-1, III d1-1-1, p0-0-1, IV d1-1-0, r0-0-1; patella I–IV 0; tibia I v2-2-2 (asymmetrical), II v1r-1r-2, III v0-0-1p, IV v0-0-1p; metatarsus I–II v2-2, III v0-0-3, IV v0-0-1p. ***Abdomen*** narrow. Dorsum with a medial pale band having scalloped edges; darker laterally.

**Female** (based on specimen AS19.0023). Carapace length 3.2, width 2.2; abdomen length 3.9. ***Carapace***: As in male, but not quite so wide, and with two fine vertical lines of pale scales on lower posterior thorax (Figs 43, 45). ***Clypeus*** narrow, dark, with black hairs. ***Chelicerae*** black to brown, with black hairs. Two promarginal and one unident retromarginal tooth, similar to those of *K.frenata* (Fig. 9). ***Legs*** honey-coloured to dark brown, first pair darker, posterior somewhat annulate. Length of femur I 1.7, II 1.5, III 1.4, IV 1.7; patella + tibia I 2.4, II 1.9, III 1.7, IV 2.6; metatarsus + tarsus I 1.5, II 1.4, III 1.5, IV 1.8. Leg spination reduced: femur I d1-1-0, p0-1-0, II d1-1-0, p0-0-1, III d1-1-1, p0-0-1, IV d1-1-0, r0-0-1; patella I–IV 0; tibia I v2-2-2 (asymmetrical), II v1r-1r-2, III v0-0-1p, IV v0-0-1p; metatarsus I–II v2-2, III v0-0-2, IV v0-0-1p. ***Abdomen*** as in male. ***Epigyne*** with openings crescent-shaped, at posterior and medial side of pale desclerotized patch.

##### Natural history.

The holotype and females from the type locality were found under bark of both small and large trees in open areas at the edge of coastal forest. Female AS19.0023 was found under bark of large tree in roadside clearing.

###### *Kelawakajufrenata* species group

The *frenata* species group includes the relatively large-bodied *K.frenata*, *K.leucomelas*, and *K.sahyadri*. They differ from other *Kelawakaju* in having pale longitudinal bands on the sides of the body, a proportionately shorter ocular quadrangle, and longer first legs in the male. The male chelicerae diverge but do not project as forward as in the *mulu* group. A narrow band of white scales descends along the front face of the male chelicerae (Figs 4, 73, 79, 83), also seen in *K.intexta*. As in *K.singapura*, the embolus is terminal on the bulb, and more or less erect, similar to those of many Dendryphantina. Two to three macrosetae on anteriolateral face of first femur are displaced ventrally and basally toward the middle of that face (as in *Padilla* Peckham & Peckham, 1894 and *Padillothorax* Simon, 1901 [Maddison et al. 2020], and more so than in *K.singapura*). *Kelawakajusahyadri* and *K.leucomelas* have been found on large trees exposed in clearings.

#### ﻿ Kelawakaju frenata

Taxon classificationAnimaliaAraneaeSalticidae

(Simon, 1901) comb. nov.

3DED9741-2F51-5921-B48E-0D7D1F0351A7

Figs 4 , 8 , 9 , 13 , 46–54 , 73–78

Ocrisiona frenata Simon, 1901.

##### Notes.

**Figures 46–72.**
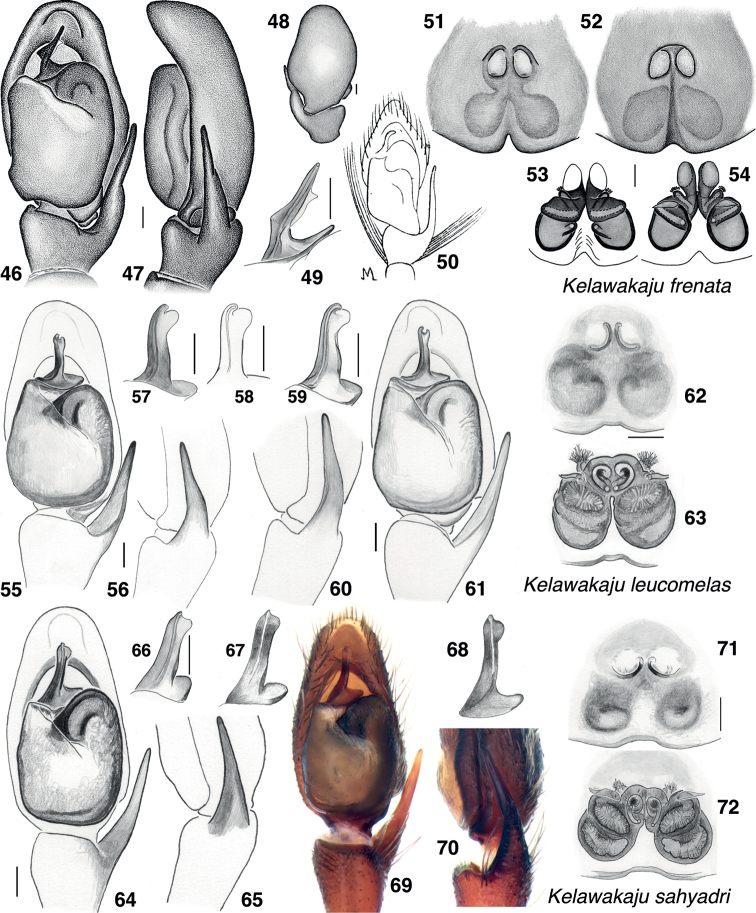
*Kelawakajufrenata* species group, genitalia **46–54***K.frenata***46** male from Dongxing, palp, ventral **47** same, retrolateral **48** same, dorsal **49** male from Hong Kong, embolus, ventral **50**Simon’s (1901a) figure, reversed **51** female d224 from Dongxing, epigyne, ventral **52** second female from Dongxing, epigyne, ventral **53** same, vulva, ventral **54** same, dorsal **55–63***K.leucomelas***55** holotype male palp, ventral **56** same, retrolateral **57** same, embolus, oblique **58** paratype male JK13.12.10.0001, embolus, oblique **59** male JK.19.08.18.0010, embolus, oblique **60** same, palp, retrolateral **61** same, ventral **62** female paratype JK.20.11.13.0001, epigyne, ventral **63** same, vulva, dorsal **64–72***K.sahyadri***64** holotype male, palp, ventral **65** same, retrolateral **66** same, embolus, oblique **67** paratype male from Kerala, embolus, oblique **68** same, ventral **69** same, palp, ventral **70** same, retrolateral **71** paratype female AS19.4934 epigyne, ventral **72** same vulva, dorsal. Oblique views of embolus are between ventral and prolateral. Scale bars: 0.1 mm.

The type specimen of *Ocrisionafrenata* Simon, 1901 has not been found, neither in the Oxford Natural History Museum (O. Pickard Cambridge collection; Simon 1901b) nor in the MNHN (Paris). Nonetheless, the application of the name is reasonably secure, as Simon’s figure (1901a: fig. 730, shown here in Fig. 50 reversed so that the right palp appears as the left) and description (1901b) match well specimens from the type locality here illustrated (Hong Kong, Fig. 49) and nearby Guangxi (Figs 46–48). Simon’s figure shows clearly the distinctive tibial apophysis of *Kelawakaju*, and the general conformation of this species group. The critical details of the embolus are unclear in Simon’s figure, and thus there remains the possibility of two very similar species at the type locality. However, at no locality have we seen two different species sympatric from the same species group, and the many photographs on iNaturalist labeled as “*Ocrisionafrenata*” from Hong Kong are credibly conspecific. Because a good case can be made for the identity of the species, and there is still hope that the type may be found, we will not designate a neotype at this time. This species was labelled “marpissine indet. [China]” in Bodner and Maddison’s (2012) molecular phylogeny; that specimen (voucher d224) was lost in the Butantan fire.

##### Diagnosis.

Differs from other *Kelawakaju* in the embolus bending suddenly toward the retrolateral, the epigynal atria with sclerotized edge both anteriorly and posteriorly (not just medially or posteriorly), and posterior notch of epigyne narrow and distinct.

##### Description.

**Male** (based on specimen from Dongxing City). Carapace length 3.1; abdomen length 4.1. ***Carapace*** dark brown, with sparse white scales. ***Clypeus*** very narrow. ***Chelicera*** dark brown, with a line of white scales on the prolateral face. One retromarginal tooth (Fig. 8). ***Palp*** dark brown, with long white scales on tibia. RTA elongate. Embolus short, from base leans slightly toward the prolateral, then twists so that its terminal part leans toward the retrolateral. ***Legs*** dark brown to yellow. First leg reddish dark brown, with sparse short white scales. Tibia with three pairs of ventral macrosetae. Legs II–IV yellow except dark brown femur, brown joints, and sparse short white scales; III and IV additionally have prolateral and retrolateral sides of tibiae and metatarsi dark brown. ***Abdomen*** dorsally cream colored, with a longitudinal, irregular, dark brown stripe, and almost entirely covered by a light brown scutum; laterally dark brown, with a pale stripe on the posterior fourth; ventrally dark brown, with a longitudinal pale stripe. Spinnerets dark brown.

**Figures 73–78.**
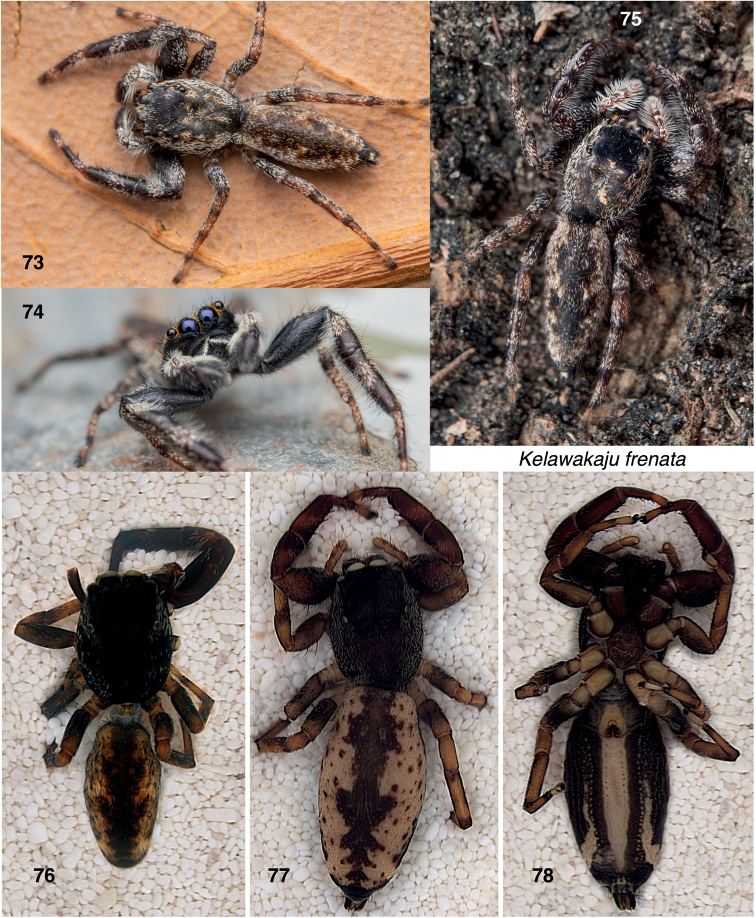
*Kelawakajufrenata***73, 74** male from Tai Tam County Park, Hong Kong (© 2020 Artur Tomaszek) **75** female from Guangdong, Gaotan Town **76** male from Guangxi, Dongxing, dorsal **77** female from Dongxing, dorsal **78** same, ventral. Specimen in **73, 74** not examined microscopically; inferred as *K.frenata* by appearance and locality.

**Female** (based on specimen from Dongxing City). Carapace length 3.45; abdomen length 5.55. Color as in male, except when mentioned. ***Chelicerae*** dark. One retromarginal tooth. ***Leg*** I light brown, with median third of femur, distal of patella and proximal and distal of tibia dark brown; tarsus yellow; II yellow, with same markings as I; III and IV as II, but with patellae entirely yellow and prolateral side of tibia dark brown. Tibia with three pairs of ventral macrosetae. ***Abdominal*** pattern as in male; no scutum. ***Epigyne*** with a pair of small copulatory openings distant from the posterior border, which has a medial excavation; internally, copulatory ducts fuse with glandular portions, spiral backwards and enter the large spermathecae, from which fertilization ducts emerge.

##### Material examined.

One male and two females in UBCZ from China: Guangxi: Dongxing City, Wanwei Village. 21.5217°N, 108.1383°E, 3 m elev., 23 May 2006, J.X. Zhang, M.S. Zhu, W.G. Lian, H.Q. Ma JXZ06#013. One male (IDWM.20018) in UBCZ from Hong Kong: Mai Po Nature Reserve, 22.2799°N, 113.9296°E, 5 July 2020, Cheuk Lun Alex Ng. One female ZRC_ENT00053870) in LKCNHM from China: Guangdong: Huidong County, Gaotan Town, Y.X. Lim, 1 October 2018. Photographs on iNaturalist suggest the species is also in Taiwan.

#### ﻿ Kelawakaju leucomelas

Taxon classificationAnimaliaAraneaeSalticidae

Maddison & Ng sp. nov.

D17BFBBF-FF65-5C8E-8C34-D2410514F2C6

https://zoobank.org/F7326873-F2B9-4DF7-8C0B-C0F6A20522B7

Figs 14 , 55–63 , 79–81

##### Type material.

***Holotype***: male (JK.20.11.13.003) in LKCNHM from Singapore: Lorong Pang Sua 1.3833°N, 103.7567°E, 13 xi 2020, J.K.H. Koh & P. Y. C. Ng. ***Paratypes***: Two females (JK.20.11.13.0001 and JK.20.11.13.0002) with same data as holotype. One male (JK.13.12.10.0001) from Singapore: Pulau Tekong, 1.4072°N, 104.0283°E, 10 December 2013, J.K.H. Koh.

##### Etymology.

Refers to the longitudinal bands of white scales (*leuco*, Greek for white) on either side of the body contrasting against the black median (*melas*, Greek for black), formed not as an adjective but more simply as the two colours themselves (and thus without an expectation of agreement with the genus name).

##### Diagnosis.

Carapace wider and ocular quadrangle shorter (Fig. 14) than in other species. Embolus differs in shape from that of the similar *K.sahyadri*: embolus tip with retrolateral flange more distinct and larger (Figs 55, 57–59, 61), and prolateral edge of embolus curves abruptly to the distal to make the embolus appear more erect (Figs 55, 61). Long brush of white hairs projecting prolaterally from male palp tibia is lacking (present in *K.frenata* and *K.sahyadri*). In the specimens we have, the body’s white side bands are more distinct than in *K.frenata* and *K.sahyadri*, and the posterior legs more uniform coloured, lacking distinct annulate markings.

##### Description.

**Figures 79–81.**
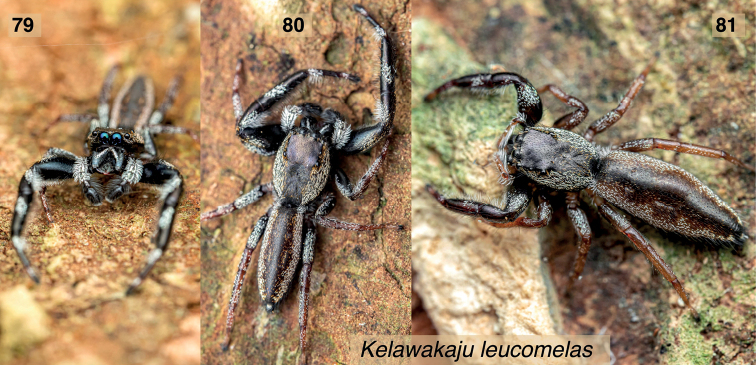
*Kelawakajuleucomelas***79–80** male from the type locality **81** female from same locality. Photographs © Chris Ang 2021. Specimens not examined microscopically; inferred as *K.leucomelas* by appearance and locality.

**Male** (based on holotype). Carapace length 3.6, width 2.6; abdomen length 4.2. ***Carapace***: Relatively flat, broad, depressed around fovea. Integument black to dark brown, clothed loosely with white scales in two broad longitudinal bands along sides, below and behind PME. Below these bands, thorax is black, without marginal white scales. ***Clypeus*** very narrow, dark, with some black hairs. ***Chelicerae*** diverging, projecting forward slightly, with a bulge anteriolaterally (as in *K.frenata*, Fig. 4, but more prominent). Bulge covered with hair-bearing tubercles. Dark brown to black, with narrow and dense line of white scales appearing as an inverted V (Fig. 79). Two promarginal and one triangular retromarginal teeth, as in *K.frenata* (Fig. 8). ***Palp*** dark brown. Patella and distal part of femur clothed with long white hairs and a few white scales. Embolus erect, with retrolateral flange separated from the tip by a distinct cleft (Figs 57–59). Endite subrectangular, with no projection, dark brown. ***Legs***: First pair dark except tarsus, slightly paler, with some patches of white scales and hairs (Fig. 79). Remaining legs medium brown, lightly dusted with white scales, without annulate markings. Length of femur I 2.7, II 1.9, III 1.6, IV 2.0; patella + tibia I 4.1, II 2.4, III 2.0, IV 2.9; metatarsus + tarsus I 2.4, II 1.6, III 1.7, IV 2.0. Leg spination reduced: femur I d1-1-0, p0-2-0, II d1-1-0, p0-0-1, III d1-1-1, p0-0-1, IV d1-1-0, 0; patella I–IV 0; tibia I v2-2-2 (asymmetrical), II v1r-1r-1p, III v0-0-1p, IV 0; metatarsus I–II v2-2, III v0-0-1p, IV v0-0-1p. ***Abdomen*** narrow and long, dark above except for band of white scales on either side, continuing the longitudinal band of the carapace (Fig. 81).

**Female** (based on paratype JK.20.11.13.0001). Carapace length 3.2, width 2.4; abdomen length 4.3. ***Carapace***: As in male, but narrower. ***Clypeus*** as in male. ***Chelicerae*** with bulge and tubercles, but less prominent than in male. Dark, with black setae. Two promarginal and one unident retromarginal tooth, similar to those of *K.frenata* (Fig. 9). ***Legs***: First pair of legs medium brown; posterior legs honey-brown, without annulate markings. Length of femur I 1.9, II 1.6, III 1.4, IV 1.7; patella + tibia I 2.7, II 1.9, III 1.7, IV 2.5; metatarsus + tarsus I 1.6, II 1.3, III 1.4, IV 1.9. Leg spination reduced: femur I d1-0-0, p0-2-0, II d1-1-0, p0-0-1, III d1-1-0, p0-0-1, r0-0-1, IV d1-1-0, r0-0-1; patella I–IV 0; tibia I v2-2-2 (asymmetrical), II v1r-1r-1p, III 0, IV 0; metatarsus I–II v2-2, III v0-0-1p, IV v0-0-1p. ***Abdomen*** narrow, long, dark medially but with pale longitudinal bands on either side. ***Epigyne*** with two crescent shaped openings posteriomedial to a pale desclerotized area (Fig. 62). (Although this specimen has the openings more medial than shown for *K.sahyadri*, another female of *K.leucomelas*, JK.20.11.13.0002, has them placed much as in *K.sahyadri*.).

**Figures 82–89.**
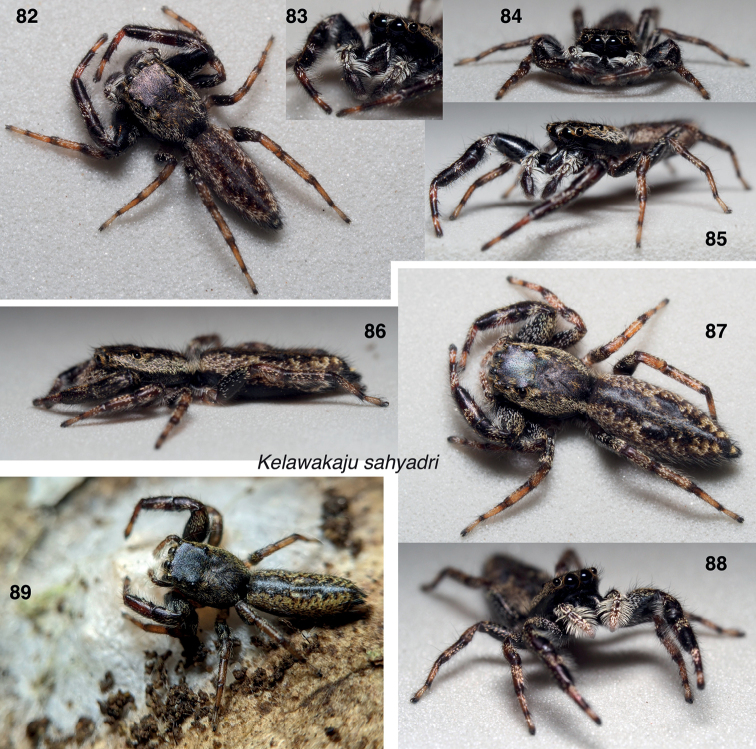
*Kelawakajusahyadri***82–85** male holotype AS19.4895 **86–88** female paratype AS19.4934 **89** female paratype from Kerala.

##### Additional material examined.

Male (JK.19.08.18.0010) in LKCNHM from MALAYSIA: Johor Bahru, Kota Tinggi, Panti Recreational Forest, 1.7872°N, 103.9425°E, 18 August 2019, P.Y.C. Ng.

##### Natural history.

Approximately ten adult and juvenile specimens were seen on tree bark at the type locality, including the holotype. The male from Kota Tinggi was found on tree bark in a sunny area near the entrance of Panti Recreational Forest.

#### ﻿ Kelawakaju sahyadri

Taxon classificationAnimaliaAraneaeSalticidae

Vishnudas, Maddison, & Sudhikumar sp. nov.

891F9188-DC92-5C25-B72D-DA3F2C3998C8

https://zoobank.org/34C05BE6-0AC9-4724-808B-1D3CC7E40610

Figs 15 , 64–72 , 82–88

##### Type material.

***Holotype***: male (AS19.4895 = NCBS IBC-BP847) in NCBS from India: Karnataka: Kodagu: Yavakapadi, Honey Valley area, 12.2224°N, 75.6553°E, 1045 m elev., 27 June 2019, W. Maddison WPM#19-083. ***Paratypes***: Female (AS19.4934 = NCBS IBC-BP848) in NCBS with data as holotype except 12.2214°N, 75.6556°E and 1130 m elev. One male and one female in CATE from India: Kerala: along state highway 21 east of Chalakudy, 10.296°N, 76.685°E, 26 June 2021, Vishnudas & Sudhikumar CATE9826705. One female with same data but 17 July 2021.

##### Etymology.

From the Sanskrit for ’from the Western Ghats mountains’, where this species lives.

##### Diagnosis.

Embolus differs in shape from that of the similar *K.leucomelas*: embolus tip with retrolateral flange less distinct and smaller (Figs 64, 66–69), and prolateral edge of embolus curves gently to the distal to make the embolus appear to be leaning slightly to the retrolateral (Figs 64, 68, 69). Compared to *K.leucomelas*, the longitudinal pale bands on body less distinct, and the carapace is narrower.

##### Description.

**Male** (based on holotype). Carapace length 3.0, width 2.1; abdomen length 3.9. ***Carapace***: Relatively flat; area around fovea slightly depressed. Dark brown, loosely clothed with white scales below and behind PLE forming an indistinct longitudinal band on each side. ***Clypeus*** very narrow, dark, with black setae. ***Chelicerae*** diverging slightly, lacking the distinct bulge of *K.frenata* and *K.leucomelas*, but with hair-bearing tubercles. Narrow stripes of white scales form inverted V as in other *frenata* group species (Fig. 83). Two promarginal and one large triangular retromarginal teeth, as in *K.frenata* (Fig. 8). ***Palp*** dark to light brown, with white scales and long white hairs. The prolateral hairs on the tibia appear as a distinct long brush projecting medially (Figs 83, 84). Embolus with prolateral edge gently curved. Retrolateral flange near tip fairly large, but emerges gradually, without strong cleft near tip (Figs 66, 67). Endite subrectangular, with no projection, dark brown. **Legs**: First leg dark to light brown, with loose patches of white setae (Figs 82, 85). Posterior legs paler, darker on femora and near the joints. Length of femur I 2.0, II 1.5, III 1.4, IV 1.7; patella + tibia I 3.1, II 2.0, III 1.7, IV 2.3; metatarsus + tarsus I 2.0, II 1.5, III 1.5, IV 1.7. Leg spination reduced: femur I d1-0-0, p1-1-0, II d1-1-0, p0-0-1, III d1-1-2, 0, IV d1-1-0, 0; patella I–IV 0; tibia I v2-2-2 (asymmetrical), II v1r-1r-1p, III 0, IV 0; metatarsus I–II v2-2, III v0-0-1p, IV v0-0-1p. ***Abdomen*** narrow, dark medially, paler and mottled laterally.

**Female** (based on specimen NCBS IBC-BP848). Carapace length 4.0, width 2.9; abdomen length 5.0. ***Carapace***, ***Clypeus*** as in male. ***Chelicerae*** dark, with black hairs arising from small tubercles. Two promarginal and one larger triangular retromarginal teeth. ***Legs***: First leg darkest, but all legs have dark patches, especially the sides of the femora and near the joints. Length of femur I 2.4, II 1.9, III 1.8, IV 2.1; patella + tibia I 3.3, II 2.5, III 2.3, IV 3.3; metatarsus + tarsus I 2.1, II 1.6, III 2.0, IV 2.3. Leg spination reduced: femur I d0-1-0, p0-3-0 or 2-0, II d1-1-0, p0-0-1, III d1-1-0, p0-0-1, IV d1-1-0, 0; patella I–IV 0; tibia I v2-2-2 (asymmetrical), II v1r-1r-1p, III 0, IV 0; metatarsus I–II v2-2, III v0-0-1p, IV v0-0-1p. ***Abdomen*** long, narrow, dark medially and pale laterally. ***Epigyne*** with two crescent-shaped openings behind a pale desclerotized area (Fig. 71).

##### Natural history.

The holotype and female paratype from Kodagu were found under loose bark of large trees standing in a field, beside a small road. The specimens from Kerala were found in crevices in the bark of *Swietenia* mahogany trees.

## Supplementary Material

XML Treatment for Kelawakaju

XML Treatment for Kelawakaju mulu

XML Treatment for Kelawakaju intexta

XML Treatment for Kelawakaju singapura

XML Treatment for Kelawakaju frenata

XML Treatment for Kelawakaju leucomelas

XML Treatment for Kelawakaju sahyadri
